# Circulating inflammatory cells in patients with metastatic breast cancer: Implications for treatment

**DOI:** 10.3389/fonc.2022.882896

**Published:** 2022-08-08

**Authors:** Caterina Gianni, Michela Palleschi, Giuseppe Schepisi, Chiara Casadei, Sara Bleve, Filippo Merloni, Marianna Sirico, Samanta Sarti, Lorenzo Cecconetto, Giandomenico Di Menna, Francesco Schettini, Ugo De Giorgi

**Affiliations:** ^1^ Department of Medical Oncology, IRCCS Istituto Romagnolo per lo Studio dei Tumori (IRST) “Dino Amadori”, Meldola, Italy; ^2^ Department of Medical Oncology, Hospital Clinic of Barcelona, Barcelona, Spain; ^3^ Translational Genomics and Targeted Therapies in Solid Tumors Group, August Pi I Sunyer Biomedical Research Institute (IDIBAPS), Barcelona, Spain; ^4^ Faculty of Medicine, University of Barcelona, Barcelona, Spain

**Keywords:** metastatic breast cancer, biomarker, inflammatory cells, NLR, prognostic, new treatments, macrophages, predictive

## Abstract

Adaptive and innate immune cells play a crucial role as regulators of cancer development.

Inflammatory cells in blood flow seem to be involved in pro-tumor activities and contribute to breast cancer progression. Circulating lymphocyte ratios such as the platelet-lymphocytes ratio (PLR), the monocyte-lymphocyte ratio (MLR) and the neutrophil-lymphocyte ratio (NLR) are new reproducible, routinely feasible and cheap biomarkers of immune response. These indexes have been correlated to prognosis in many solid tumors and there is growing evidence on their clinical applicability as independent prognostic markers also for breast cancer.

In this review we give an overview of the possible value of lymphocytic indexes in advanced breast cancer prognosis and prediction of outcome. Furthermore, targeting the immune system appear to be a promising therapeutic strategy for breast cancer, especially macrophage-targeted therapies. Herein we present an overview of the ongoing clinical trials testing systemic inflammatory cells as therapeutic targets in breast cancer.

## Introduction

Over the past years, the role of the immune system in cancer development and progression has gained increasing attention. The immune system has a paradoxical behavior during cancer development; some immune cells are able to recognize tumor cells and defend the host (immunosurveillance), whereas other cells can contribute to activating immune escape mechanisms (1). A condition of persistent smoldering inflammation, determined by oncogenic mutations in tumors, creates an inflammatory microenvironment typical of cancer tissue (2). This “low grade” inflammation leads to the proliferation and survival of malignant cells, promotes angiogenesis, subverts adaptive immune responses and leads the immune cells towards an immunosuppressive phenotype (3). Tumor-associated chronic inflammation is definitely a hallmark of cancer that fosters progression to a metastatic stage (4).

Immune cells in tumor microenvironment (TME) and in the peripheral blood are significantly involved in breast cancer (BC) diffusion (5). Circulating inflammatory cells are characterized by pro-tumor activities such as enhanced angiogenesis, chemokine production or immune-surveillance and promote the metastatic potential of tumor cells (6).

BC is linked to modifications in systemic inflammatory indexes. Platelet, neutrophil, lymphocyte, monocyte counts, inflammatory cytokines and acute phase proteins (like C-reactive protein or PCR) are considered potentially new prognostic parameters. Combined indexes have been determined to define the condition of systemic inflammation as the platelet-lymphocytes ratio (PLR), monocyte-lymphocyte ratio (MLR), neutrophil-lymphocyte ratio (NLR) and systemic immune-inflammation index (SII) (7). These lymphocyte indexes have been correlated to prognosis in many solid tumors and are considered applicable in clinical practice as reliable independent prognostic markers (8–12).

It is plausible that imbalances in the ratio of immune cellular counts may provide an insight into underlying tumor progression and prognosis also in patients with BC. The availability and non-invasive nature of these indexes makes them affordable biological markers.

One of the major questions is whether cancer-related inflammation can be exploited into useful approaches in treating advanced/metastatic BC (aBC).

In this review, we will provide an overview of the potential prognostic value of lymphocytic indexes in aBC and discuss the therapeutic potential of targeting the immune system in this context.

## Circulating inflammatory cells and prognosis in BC

### Platelets, neutrophils and lymphocytes

**Figure 1:**
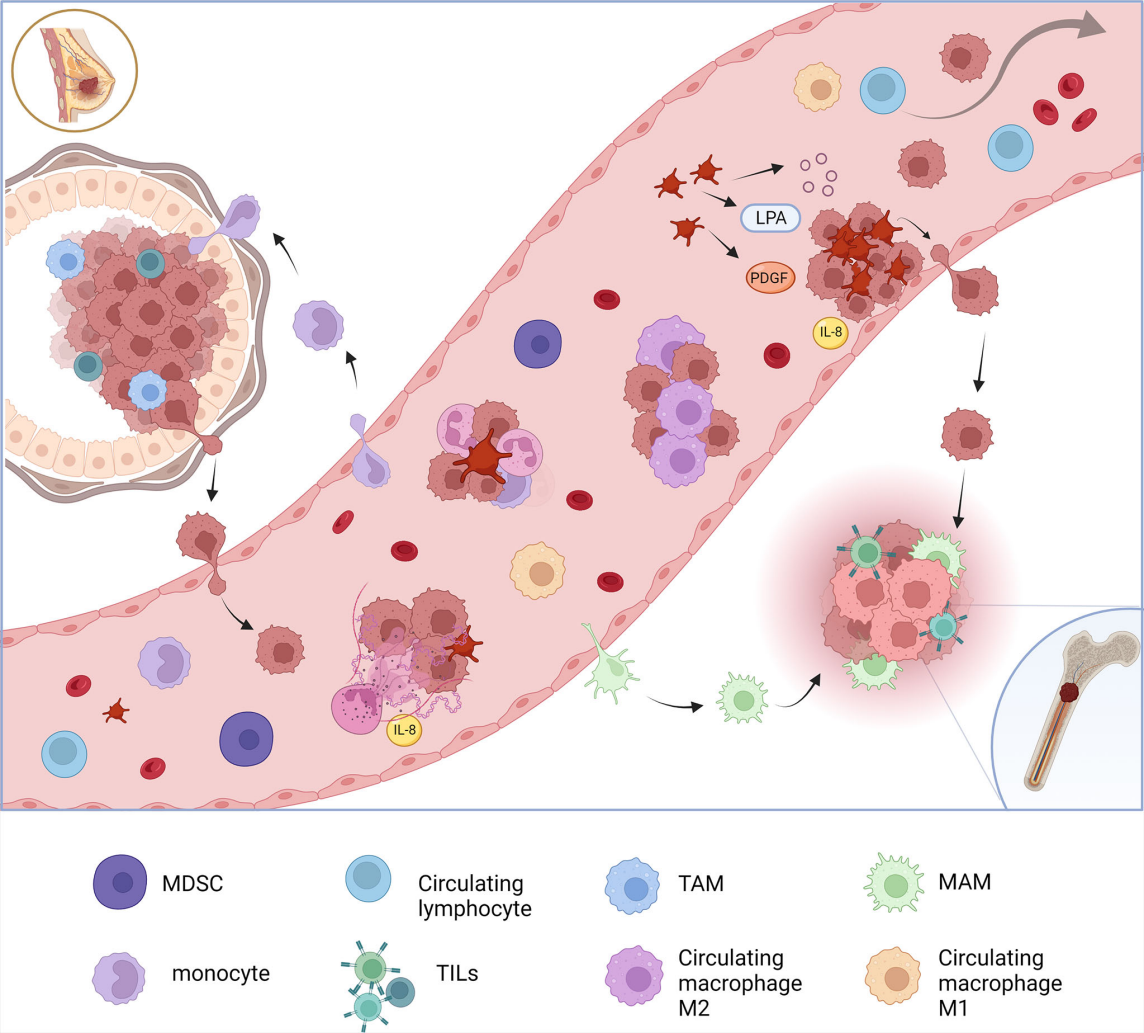
Circulating inflammatory cells in blood flow in breast cancer. Inflammatory cells are involved in many ways in promoting cancer cells invasiveness. Evasion of tumor cells from the primary site into circulation is partially permitted by tumor associated macrophages and other immune cell responsible for an immunouppressive microenvironment. Immune cells are also attracted by tumor factors from the blood flows. Circulating tumor cells (CTCs) in blood flow are accompanied in cluster with macrophages and monocytes. Neutrophils release neutrophils extracellular traps (NETs) that determines aggregation of CTCs and other immune cells guaranteeing their survival and a favorable microenvironment in circulation. Platelets, activated by tumor promoting factors, trigger hemostasis mechanisms that catch CTCs cells favoring the adhesion to vessel walls. Lysophosphatidic acid (LPA) dependent mechanism, platelet derived growth factors (PDGFs), interleukin-8 (IL-8) and platelet-derived extracellular vesicles (PEVs) contribute to the formation of platelet clots that include and protect CTCs. CTCs that are not included in aggregates are unlikely to survive in the bloodstream. Immune cells are also important in the formation of the metastatic-niche. Macrophages associated to metastasis (MAMs) derive from the bloodstream and are recruited in the process of metastasis. Adapted from “Breast Cancer to Brain Metastasis”, by BioRender.com (2022). Retrieved from https://app.biorender.com/biorender-templates.

Platelets have a crucial role as regulators of inflammation and are involved in various stages in BC development and dissemination (13). Tumor-activated platelets further contribute to cancer progression by promoting critical processes such as angiogenesis and metastasis. Platelets modulate innate immunity (antigen presentation by dendritic cells, monocyte recruitment and differentiation or neutrophil extracellular trap formation) and also promote thrombosis and metastasis (for example with the mechanism of lysophosphatidic acid-dependent (LPA) metastasization or formation of platelet clots) (**Figure 1**) (14). Moreover, the adaptive immune responses can be modulated also by platelets inducing the differentiation of T-helper 17 cells (13).

Platelet-derived growth factors (PDGFs) contribute to sustaining proliferative signals. Among them we recognize PDGF, transforming growth factor –beta (TGF-beta) and platelet -derived endothelial cell growth factor (PD-ECGF) that are often produced by BC cells and enhance their progression and aggressiveness (15). Platelet-derived extracellular vesicles (PEVs) are also considered potential mediators in the activation of signaling connected to migration in metastatic BC cell lines (16).

Furthermore, the link between hemostasis and BC assumes that platelets have a central role in disease progression (17). In peripheral blood, tumor cell interaction with adherent platelets arrest tumor cells thanks to adhesion proteins and crosslinking plasma protein ligands that support platelets to adhere to the vessel wall. Tumor cells that fail to attach are rapidly cleared from the circulation and undergo apoptosis. To facilitate adhesion to platelets, some cancer cells can upregulate aberrant surface proteins. The binding to platelets helps metastatic cells to arrest within the microvessels of their target organs, where then they extravasate, start to proliferate at the attachment site, or remain dormant for extended periods of time (18). PDGFs support the proliferation and extravasation of invading metastatic cells in the metastatic niche (**Figure 1**) (19).

Furthermore, BC cells secrete high levels of interleukin-8 (IL-8) in response to platelets that may activate their AKT pathway promoting an invasive capacity. Patients with BC receiving aspirin had lower circulating IL-8, and their platelets did not increase tumor cell invasion compared with patients not receiving aspirin (20, 21).

In BC, elevated platelet-related markers may be associated with poor prognosis. The meta-analysis of 17,079 individuals conducted by Guo et al. confirms that an high PLR is associated with poor overall survival (OS) as well as high risk of recurrence for BC patients (22). However the metanalisis includes few studies about aBC patients, therefore the specific relationship between PLR and aBC need to be better explored.

Neutrophils have a central role in inflammatory response; patients with various cancer types, including BC, often exhibit increased numbers of circulating neutrophils (9).

Neutrophils with an immature phenotype have been observed in the blood stream of cancer patients. Increased levels of tumor-induced granulocyte-colony stimulating factor (G-CSF) and granulocyte–macrophage-colony stimulating factor (GM-CSF) enhance hematopoiesis towards the production of myeloid cells, granulocyte–monocyte progenitors (GMPs) and neutrophil progenitors (23).

Neutrophils seem to be involved in BC progression promoting metastasis-initiating cells that drive cancer spread (24, 25). They can secrete immunosuppressive mediators and angiogenic factors such as reactive oxygen species, vascular endothelial growth factor (VEGF) and matrix metalloproteinase 9 (MMP-9) contributing to a pro-tumor microenvironment (26). Neutrophil-secreted factors alter the heterogeneity of cancer cells, favoring breast metastasis-initiating cells (27). In a BC model, neutrophils induced by tumor cells showed to suppress CD8+ T lymphocytes promoting metastasis through immunosuppression (28). Furthermore, it has been observed that neutrophils may support the metastatic potential of circulating tumor cells (CTCs) in patients with metastatic disease (29).

In BC, the formation of neutrophils extracellular traps (NET), web-like structures formed by DNA and intracellular contents expelled by these cells, has been linked to increased invasiveness and risk of venous thromboembolism (30). The tumor releases pro-inflammatory factors, pro-NETotic factors and extracellular vesicles into the circulation that can activate platelets and the endothelium causing NET release. NETs can capture CTCs, promote the formation of metastases and also the extravasation in the damaged endothelium and generate a highly inflammatory microenvironment for the pre-metastatic niche (**Figure 1**) (30). NETosis seems to be more frequently produced by morphologically circulating immature neutrophils that express a pro-metastatic behavior, as observed in an *in vitro* model of BC liver metastasis (31). IL-8 is able to cause neutrophils NETs release and at the same time has an important chemoattractant effect for these cells in the BC microenvironment (32).

NLR is the most widely evaluated inflammatory index. Its elevation is associated with poor prognosis in several cancers and showed to be an independent factor of outcome prediction (9, 33). The prognostic value of the NLR index has been studied in BC (34). A systematic review of fifteen studies analyzing a total of 8563 patients highlighted that a high NLR is associated with a poor OS and DFS in patients with BC especially in triple negative disease and HER-2 positive (HER2+) BC population rather than hormone receptor-positive (HR+) BC patients (35). In a retrospective study, that had the aim to determine the prognostic implications of NLR in the peripheral blood of patients with malignant bone metastasis collected from a prospective cohort, the ratio was significantly associated with tumor type (P<0.0001, included BC) (36).

The combination of NLR/PLR can be considered a more stable marker to changes as compared to single ratios, which may be influenced by concomitant drugs or conditions (e.g. infections or corticosteroids). Combined indexes may reflect also the immune balance and the patients’ immunogenic phenotype as a worse independent prognostic indicator from common prognostic factors such as grading, Ki-67, and molecular subtypes (37). However, conflicting data persist regarding the utility of NLR in predicting prognosis in patients with metastatic disease.

Similarly to NLR and PLR, lymphocyte-to-monocyte ratio (LMR) reflects the imbalance between adaptive and innate immune system in patients with advanced neoplasia and with an inadequate anti-tumor activity (38–40). Lower LMR has been associated with poor survival in BC (41–44). Few studies showed how lymphopenia can be a predictor of poor outcome in aBC patients with increased risk of disease progression and worse long-term survival, assuming a link to a weak anti-tumor response and lower tumor-infiltrating lymphocytes (TILs) (45, 46).

TILs are an emerging tissutal predictive biomarker for BC and their phenotype influences the TME. Infiltration of type 2 (CD4+ T-helper cells or Th2), including Forkhead box P3 (FOXP3) CD4+ regulatory T-cells, inhibits cytotoxic T-cells (CTL) function, supports proliferation and promotes an adaptive anti-inflammatory immune response that is responsible for tumor growth. Especially TNBCs may present a lymphocytic infiltrate >50% and are consequently termed “lymphocyte predominant BCs” (47).

The circulating lymphocyte count and lymphocytes characteristics, especially T-cell receptor diversity, have been investigated, either alone or in combination, as prognostic factors at diagnosis in aBC patients (48). It was observed that the severe restriction of TCR diversity (≤ 33%) was independently associated with shorter OS (48). In addition, the quantitative alteration of lymphocytes in the peripheral blood, and mainly the CD4+ lymphopenia, resulted to be strongly associated with aBC progression (46). In a study from Trédan et al., the cohort of patients with aBC treated in first line showed a median OS of 1.2 months for severe CD4+ lymphopaenic patients, 14.7 months for patients with mild CD4+ lymphopaenia and 24.9 months for non-CD4+ lymphopaenic patients (log rank p-value < 10−4) (46). Importantly, the relative majority of immunosuppressive cytokines (IL-6 and IL-10) and immunosuppressive circulating lymphocytes, like CD8+CD28- suppressor T lymphocytes, in peripheral blood of aBC patients have been associated with a shortened PFS (49).

## Circulating myeloid suppressor cells and macrophages

Immune myeloid cells, such as myeloid-derived suppressor cells (MDSCs), macrophages and monocytes also showed to play a major role in BC. Tumor-induced systemic immune changes might be reflected by some peripheral blood immune cells alterations (50). For example, monocytes are attracted to tumors by many chemokines and motility factors released by the same BC cells, including interferon-γ (INFγ) (51), and lower IFNγ signaling responses in peripheral monocytes tend to correlate to an increased tumor macrophages infiltration.

Circulating monocytes are recruited at the tumor level and induced to differentiate into macrophages that have a central role in the TME. These cells are also directly associated with CTCs in peripheral blood of aBC patients, especially in TNBC (52), and might be involved in guiding CTCs migration in the peripheral circulation to the metastatic niches (**Figure 1**) (53).

An imbalanced ratio between monocytes and lymphocytes (MLR) underlines the alteration in immune defense against cancer evasion. In a study involving more than 500 patients with aBC, among various immune indexes, only MLR was able to independently predict OS, especially in TNBCs, implying a substantial difference between biological subtypes (52). In the same study, among other predictors of the outcome, CTC (≧̸5 versus <5), metastatic sites, and tumor subtypes (TNBC versus HER2-/ER+ tumors) remained significant. However, several unanswered biological questions remain, such as what determines the tropism of these inflammatory cells or CTCs at a specific metastatic site (e.g. bone) (54) and, in TNBC, which biological characteristic and which different treatment could have a major impact on the metastatic potential of these single cells (55, 56). Another study corroborated these findings showing in the univariate analysis that MLR-high patients with aBC experienced poor prognosis (HR 1.77, 95% CI: 1.24–2.54, p=0.002) (57). MLR was also significantly associated with the extension of the metastatic disease at presentation. The prognostic impact has been also evaluated analyzing the variation of MLR (and also PLR, NLR) during treatment. The reduction or stability of the ratios was associated to better OS (MLR p = 0.028, NLR p = 0.034 and PLR p = 0.003) (57).

The outcome of metastatic BC seems to be also affected by the type of circulating macrophages. Aberrant macrophage polarization has been observed in BC patients. Polarized macrophages are usually classified as M1 or M2 macrophages. M1 subtypes are characterized by intracellular killing and tumor resistance. M2 macrophages instead are associated with immunosuppressive phenotype and are further categorized into other three subtypes: M2a, induced by interleukin-4 (IL-4) or interleukin-13 (IL-13); M2b, induced by immune complexes and agonists of toll-like receptors or interleukin-1 receptors (IL-1R); and M2c, induced by interleukin-10 (IL-10) and glucocorticoid hormones (58). M2a macrophages, differentiated *in vitro* with IL-4/IL-13, significantly increase the migratory and invasive potential of BC cells compared to M2b or M2c macrophages (59). Some studies observed that the percentages of M2-macrophages are high in BC patients, especially a higher percentage of M2c subtype was observed in patients with advanced disease, highlighting the role of IL-10 in facilitating tumor progression (60). The M2 population has also been associated with clinical parameters such as lymph node metastasis, advanced stages, histological differentiation (p<0.05). The authors also observed that ER negative (ER-) patients show higher levels of M2-like monocytes (61).

The importance of phenotype of circulating monocytes has also been highlighted by high gene expression of MMP-1 and MMP-11 in peripheral mononuclear cells of BC patients correlating to an increased hematogenous diffusion stimulated by interaction with BC cells and cancer associated fibroblasts (CAF) (62).

Analyzing the specific monocyte sub-populations has defined also a link between high levels of systemic CD14+CD16++ monocytes and better OS and PFS in ER-positive and ER-negative BC patients respectively (63). This suggests the potential therapeutic targeting of circulating immune cells.

In TME, tumor-associated macrophages (TAM) are involved in advanced tumor development, progression and dissemination. They contribute to matrix specific formation or degradation and immunosuppression (64). Tumor derived stimuli (anti-inflammatory cytokines IL-4, IL-10, IL-13 and TGF-B), contribute to polarizing TAMs toward an immunosuppressive function as observed in peripheral blood. M2-macrophages increase the expression of specific receptors (some of them are CD68, CD163, CD206, CD204 and macrophage receptor with collagenous structure or MARCO), and the production of VEGF and IL-10, favoring an immunosuppressive environment (64, 65).

Macrophages can also be differently influenced by various breast tumor histotypes due to a specific crosstalk between them and cancer cells. The TNBC-educated macrophages down-regulate citrulline metabolism and differentiate into M2-like macrophages with increased macrophage mannose receptor (MMR) expression, a commonly used marker to define M2 (66). In the TME macrophages enhance the inhibition of T cell response and the recruitment of immunosuppressive leukocytes reducing the tumoricidal function. Macrophages promote angiogenesis (through the secretion of VEGF by perivascular TAMs) and the production of matrix metalloproteinase (MMP) enzymes that remodel the tumor stroma facilitating migration and intravasation (64). In inflammatory BC (IBC), TAMs contribute to its metastatic phenotype, due to a production of cytokines (IL−6, IL−8, and IL−10) that are sufficient to develop the migration effect. In IBCs cells the Ras homology GTPase RhoC is necessary for the enhanced migration response after TAMs signals (67). In general a high infiltration of TAMs is associated with unfavorable features in patients with aBC.

Macrophages also work on the tumor cell seeding of metastatic sites, constituting metastasis associated macrophages (MAMs). Measurements of the monocyte trafficking from TME in a metastatic BC preclinical mouse model showed that MAMs are derived from inflammatory monocytes that are specifically early recruited in the process of pulmonary metastasis, before other immune cells and resident macrophages (68). The recruitment of inflammatory monocytes, which express CCR2 (the receptor for chemokine CCL2), as well as the subsequent recruitment of MAMs, is dependent on CCL2 synthesized by both the tumor and the stroma (69). MAMs are abundant in BC bone metastases (prevalent form of metastasis in BC patients) (70) and derive in large part from recruited inflammatory monocytes. The recruitment of these cells is mostly mediated by the CCL2-CCR2 signaling and CSF1-CSF1 receptor pathways, which are critical for BC metastasis outgrowth and are considered a potential new therapeutic target (71).

The presence of TAMs has been associated with resistance to classical treatments in BC. TAM-mediated chemoresistance has been observed preclinically after paclitaxel infusion. The high recruitment of TAMs due to the CSF1-CSF1R signaling suppresses the mitotic-arrest induced by the taxane (72, 73). It has also been observed resistance to immunotherapy and anti-HER2 agents, especially due to the ability of TAMs to reduce the presence of cytotoxic lymphocytes (74).

Myeloid-derived suppressor cells (MDSCs) are commonly related with tumor progression, angiogenesis and poor prognosis in different cancer types, due to their capacity to elude immune-surveillance. MDSCs are a heterogeneous group of immature myeloid cells (IMCs) with strong immunosuppressive patterns and functions. In physiological conditions, IMCs quickly differentiate into mature leukocytes which play essential roles in host defense against pathogens (75). However, in some conditions such as cancer or inflammation, IMCs fail their normal differentiation and acquire the features of an immature and dysfunctional myeloid population, namely MDSCs with the capacity to suppress cytotoxic T cell responses (76). According to surface antigen expression, MDSCs can be differentiated in granulocytic-MDSCs (G-MDSCs; including neutrophils, eosinophils, basophils, and mast cells) and monocytic-MDSCs (Mo-MDSCs; including monocytes, macrophages, and dendritic cells) (77, 78).

In BC patients, MDSCs seem to be enriched in peripheral blood and can correlate with a poor prognosis, clinical stage and metastatic extension (79, 80). The enrichment of MDSCs is related to an immunoregulatory switch that facilitates the transition to a systemic and more aggressive disease (81). Bergenfeltz et al. observed that an increased level of Mo-MDSCs is detectable in peripheral blood of aBC patients (82). A study by the same authors shows how high levels of Mo-MDSCs are significantly associated with ER- tumors, disease progression, worse progression-free survival, liver and bone metastasis. The inflammatory stimuli, typical of ER- BC (as GM-CSF produced by tumor cells), induces Mo-MDSCs accumulation (83). The same study observed an interesting association between MDSCs and CTCs, supposing a possible clusterization of CTCs with leukocytes including MDSCs capable of enhancing tumoral cells dissemination and metastasization. Besides their known immunosuppressive functions, MDSCs also have direct effects on BC cells contributing to invasiveness and metastasis through the activation of the intracellular phosphatase and tensin homolog (PTEN)/Akt pathway that results in an increased expression of MMP and promotion of invasion and metastasis (75). The phosphoinositide 3-kinase gamma (PI3K γ) signaling plays a crucial role in the activation and migration of myeloid cells, and its expression in MDSCs facilitates tumor growth (84).

The wide involvement of MDSCs, macrophages and monocytes in the mechanisms of BC progression makes them an interesting biomarker to be studied in depth as a potential therapeutic target. Prospective studies are required to define the real effectiveness of circulating inflammatory biomarkers in aBC (**Table 1**).

**Table 1:** Lists of various potential new biomarkers and implication in clinical practice.

Biomarkers in aBC	Potential use in clinical practice	References
PLR	High PLR correlate to worse OS	(22)
NLR	High NLR correlate to worse OS and DFS	(34, 35)
MLR	High MLR correlate to worse OS (especially in TNBC) (*p* = 0.013^51^, p= 0.002^56^)	(52, 57)
Lymphopenia	Predictor of increased risk of progression and worse OS	(45, 46)
Pro-tumor circulating macrophages	M2 in blood of BC patients are associated with advanced stages	(60)
MDSCs	Enriched MDSCs in blood of BC patients can correlate with poor prognosis and metastatic extension	(79, 80)

M2, pro-tumors macrophages; MDSCSs, myeloid-derived suppressor cells; PLR, platelet-lymphocytes ratio; MLR, monocyte-lymphocyte ratio; NLR, neutrophil-lymphocyte ratio; OS, overall survival; DFS, disease free survival.

## Immune circulating biomarkers and prediction of response to treatments in aBC

In the metastatic setting, more predictive markers for therapeutic efficacy, as well as prognostic biomarkers, are urgently needed.

High NLR, MLR and PLR showed a significant association with shorter progression free survival (PFS) in metastatic ER- BC patients treated with eribulin based regimen hypothesizing that the histological subtype and high NLR (the only independent factors at the final analysis) might be related to low responsiveness to this treatment (85). NLR and PLR are also predictive of benefit from platinum-containing chemotherapy specifically in metastatic TNBC patients. In the study conducted by Vernier et al. patients receiving carboplatin based chemotherapy with higher PLR and NLR experienced a worse PFS compared to ER+/HER2− patients treated with the same regimens (86). These feasible indexes could also be combined with germline or somatic BRCA 1/2 gene mutation and TILs that are actually considered strong predictive and prognostic biomarkers in TNBC (87). Further research is needed to evaluate a potential correlation existing between these biomarkers.

NLR and PLR may also represent a predictive marker for response to endocrine therapy in stage IV BC (12, 88, 89). Lymphocytic indexes have been studied in patients with ER+ aBC in correlation to response to new treatments with contrasting results. To date, Cyclin Dependent Kinase 4 and 6 (CDK 4/6) inhibitors are the first line treatment for this histological subtype associated with aromatase inhibitors or fulvestrant, and preclinical evidence indicates that these new treatments have the ability to stimulate antitumor immunity (90). A retrospective study showed an independent association between high NLR or PLR and lower PFS after three cycles of CDK4/6 inhibitors treatment (p = 0.007 and p = 0.005, respectively) (91). Also Weiner et al. at SABCS 2020 presented a study where PLR at baseline resulted to be associated with worse PFS of patients treated with first line CDK4/6 inhibitors (92). The same association between NLR and PFS has been observed in a retrospective study involving patients treated with everolimus-based treatments (p=0.01) (12).

The impact of lymphocytic indexes was also evaluated in HER2+ aBC patients receiving dual anti-HER2 blockade. In a cohort of 57 patients only the Pan-Immune-Inflammatory Value (PIV), (defined as the product of peripheral blood neutrophil, platelet, and monocyte counts divided by lymphocyte counts) was statistically significantly associated with worse OS at multivariable analysis (93). In the same population, the single indexes (MLR, NLR, and PLR) did not demonstrate a significant association to prognosis, but correlated with worse outcomes. The effects of these monoclonal antibodies might be mediated by systemic peripheral inflammatory cells, especially circulating lymphocytes, in association to TILs present in the TME (94).

There is an urgent need to identify effective biomarkers for predicting survival benefits from ICIs in patients with TNBC after the demonstration of the efficacy of atezolizumab and pembrolizumab in this category of patients (95). The 20% of this BC histological subtype expresses Programmed cell Death protein-1 (PD-1), an immune checkpoint receptor that limits T-cell effectors function within tissues interacting with its specific ligand PD-L1 (96). PD-L1 is expressed on the membrane of BC cells and recognized by the specific receptor on CD8 + T cells. Immune checkpoint inhibitors (ICIs) may influence systemic inflammation in patients and in conditions of low lymphocyte counts the efficacy of these drugs may be invalidated (97). Studies among various malignancies (including aBC) demonstrated that higher NLR is significantly associated with poorer OS and PFS, lower rates of response and clinical benefit (26). The combination of NLR and tumor mutational burden (TMB) increased the capacity of predicting the outcome after an ICIs treatment; indeed, the category of patients with NLR-low/TMB-high showed higher response rate (26).

Although the increasing evidence available suggests a relationship between lymphocytic ratios and prognosis in aBC (**Table 2**) several issues persist about the feasible clinical application. First of all there is lack of consensus regarding a shared cut-off value, secondarily the sensibility and specificity of these ratios varies among different studies and almost the totality of the studies are retrospective. Finally large prospective studies with a rigorous methodology are mandatory to determine the real clinical value and applicability of inflammatory indexes.

**Table 2:** Prognostic role of circulating biomarkers in response to treatments in aBC, available results from retrospective analysis.

BC subtypes	Treatment	Biomarker	Outcome	References
HR+ BC	cdk4/6 inhibitors	High PLR, High NLR	Poor PFS (PLR p = 0.007, NLR p = 0.005 respectively)^90^; (high PLR at baseline p=0.04)^91^	(91, 92)
	everolimus-exemestane	High NLR	Poor PFS (p = 0.01)	(12)
HER2+ BC	P+H+ chemotherapy	High PIV	Poor OS (*p* = 0.002)	(93)
TNBC	chemotherapy platinum based	High PLR High NLR	Poor PFS (p < 0.001)	(86)
ER-	Chemotherapy (eribuline)	High NLR	Poor PFS (p= 0.003)	(85)

P+H, pertuzumab + trastuzumab; PIV, Pan-Immune-Inflammatory Value (defined as the product of peripheral blood neutrophil, platelet, and monocyte counts divided by lymphocyte counts).

## Systemic inflammatory cells as therapeutic target or vehicle of treatment

Circulating inflammatory cells are considered a useful target in the therapeutic strategy for aBC due to their pro-tumor involvement. However, the delicate balance between the tumor-inhibitory and tumor-promoting properties of immune cells implies the need for adequately targeted therapeutic approaches.

### Targeting platelets

The clinical benefit of targeting tumor-cell platelets interaction in aBC is still under question. Many studies support the idea of utilizing targeted platelet therapies to inhibit the platelet’s role in the malignancy. Platelets exposed to tamoxifen or ticagrelor release significantly lower amounts of pro-angiogenic VEGF and have less interaction with BC cells (98, 99). However the concomitant use of anti-platelet therapy in cancer patients has a rationale but carries many risks as the declining platelet function and counts as a consequence of disease progression or myelosuppressive effects of treatments.

### Targeting peripheral neutrophils and TANs

As previously reported, TANs are particularly involved in tumor progression and studies on new drugs are evaluating therapeutic strategies on several fronts: inhibition of neutrophils recruitment in tumors, depletion of neutrophils in TME, targeting tumor-promoting TAN polarization (100). Targeting neutrophils as a treatment option has been investigated in many preclinical models with discouraging results due to the short life span of these cells (nearly 24 h in blood) (101). Low toxicities strategies to inhibit protumor neutrophils are warranted and expected as promising approaches. Treatments that target the mechanism of interaction between tumor cells and neutrophils are more encouraging.

INF-β and TGF-β are cytokines with a role in switching neutrophils polarization from N1 to N2. In a BC mouse model the blockade of TGF-β increased the percentage of N1 and the activity of CD8+ T cells (102). A phase I trial enrolling patients with solid tumors (including BC patients) has the objective to evaluate the efficacy of a selective and orally active TGF-β receptor 1 inhibitor (NCT03685591). This new TGF-β receptor 1 inhibitor combined with palbociclib in a xenograft BC model led to a significant increase in OS, suggesting the potential for such combination (103).

Neutrophils are considered the major productors of pro-angiogenic factors and the presence of a rich neutrophils infiltrate in TME has been associated with resistance to anti-VEGF therapies (104). Tumors enriched in neutrophils are also more likely resistant to ICIs. Consequently, there are many ongoing studies (phase I/II) evaluating the association between ICIs and new compounds against neutrophils in solid tumors, but results are still awaited (**Table 3**) (104). Chemokines and interleukins involved in the TAN recruitment (like CXCL1, 2, 5, 6, 8, IL-6, IL17) and their signaling are possible new targets for inhibitory drugs associated with ICIs enhancing their activity (105). The inhibition of enzymes involved in the protumor phenotype as nicotinamide phosphoribosyl transferase (NAMPT) or CXCR2 signaling resulted in an effective reduction of tumor growth and polarization to N1 (106).

**Table 3:** Clinical trials with new treatment targeting neutrophils and MDSCs.

Target	Drug	Concomitant drugs	Clinical trial	Histology	phase	status
IL1-β	Canakinumab	Spartalizumab, LAG525, NIR178, Capmatinib,MCS110	NCT03742349	aTNBC	I	recruiting
LXR-α/β	RGX-104	Nivolumab, ipilimumab, pembrolizumab	NCT02922764	aST	I	recruiting
	MV-s-NAP		NCT04521764	aBC	I	recruiting
ARG1	INCB001158	Pembrolizumab	NCT02903914	aST	I/II	recruiting
NOS	L-NMMA	Pembrolizumab, IL-12 gene therapy, Docetaxel	NCT04095689	eTNBC	II	Suspended (protocol revisions, waiting for approval)
TGF-β R1	PF-06952229		NCT03685591	aST	I	recruiting
TAM receptors	Sitravatinib		NCT04123704	aBC	II	recruiting
SIRPα	TTI-621	Pembrolizumab	NCT02890368	aST	I	no result posted, terminated
HDAC	Entinostat	Ipilimumab, Nivolumab	NCT02453620	aBC	I	Active, not recruiting
HDAC	Entinostat	Exemestane, Goserelin Acetate	NCT02115282	HR+ aBC	III	Active, not recruiting
HDAC	Entinostat	atezolizumab	NCT02708680	aTNBC	I	unknown
HDAC	Entinostat	Ipilimumab, Nivolumab	NCT02453620	aST	I	Active, not recruiting
HDAC	Entinostat	capecitabine	NCT03473639	eBC	I	recruiting
PI3K-γ	Eganelisib	Bevacizumab, Atezolizumab, Nab-paclitaxel	NCT03961698	aTNBC, RCC	II	recruiting
	tenalisib		NCT05021900	aBC	II	recruiting
	copanlisib	pertuzumab, trastuzumab	NCT04108858	aBC	I/II	recruiting
C/EBPα	MTL-CEBPA	pembrolizumab	NCT04105335	aST	1a/1b	recruiting
IRE1	ORIN1001	abraxane	NCT03950570	aBC	I/II	recruiting
IL-6	sarilumab	Capecitabine	NCT04333706	aBC	I/II	recruiting

aBC, advanced Breast cancer; eBC, early Breast cancer; aST, advanced Solid Tumors; aTNBC, advanced Triple Negative Breast Cancer; HR+ aBC, hormone receptor positive Breast Cancer, RCC, Renal Cell Carcinoma; HDAC, Histone deacetylases; IL-6, interleukine-6; IRE1, inositol-requiring enzyme 1; C/EBPα, CCAAT-enhancer-binding protein alpha; PI3K-γ, phosphatidylinositol 3-kinase gamma; SIPRα, signal regulatory protein alpha; TAM receptors, TYRO3/AXL/MERTKM; TGF-β R1, Transforming growth factor beta receptor one; NOS, Nitric oxide synthases; ARG1, arginase protein 1; LXR-α/β, liver x receptor-alpha/beta; IL-1β, Interleukin 1 beta.

### Targeting MDSCs

Targeting MDSCs may also become a potential strategy to enhance antitumor activity of current treatments (**Table 3**). Entinostat, a selective HDAC1/3 inhibitor, can decrease the populations of MDSCs and FOXP3+ Tregs in murine models of mammary carcinoma (107). Combination of entinostat with nivolumab and ipilimumab is currently under evaluation in a phase I trial in patients with invasive and metastatic BC (NCT02453620) (**Table 1**). Entinostat showed promising preclinical and clinical data in HR+ endocrine-resistant BC. G-MDSCs and Mo-MDSCs manifested a reduction (14.67 *vs* +20.56%; p = 0.03 and -62.3 *vs* +1.97%; p = 0.002 respectively) in a *post hoc* analysis of samples from entinostat treated patients in ENCORE301 trial (108). CD40 was also significantly downregulated in the majority of MDSC subsets (109). Other drugs like IPI-549 (eganelisib) transported by liposomes, can inhibit PI3Kγ in MDSCs, resulting in downregulation of arginase 1 (Arg-1) that conduces to MDSCs apoptosis and reduction of their immunosuppressive activity to CD8+ T cells. This strategy synergizes with ICIs and inhibits tumor growth *via* facilitating the dendritic cell maturation and tumor infiltration of CD8+ T cells while decreasing the tumor infiltration of immunosuppressive regulatory T cells, MDSCs, and M2-like TAMs in solid tumors (81). Some trials targeting PI3Kγ involving BC patients are currently ongoing (**Table 3**).

Sitravatinib is an oral spectrum-selective tyrosine kinase inhibitor that targets the TYRO3/AXL/MERTK pathways and split the VEGFR2/KIT family receptor tyrosine kinases (RTKs). Inhibition of this pathway may promote the depletion of MDSCs in the TME and at the same time repolarize TAMs towards the M1 phenotype (110). The NCT04123704 trial is evaluating sitravatinib in aBC.

The myeloid lineage in solid tumors can also be targeted by MTL-CEBPA, a novel immunotherapy constituted by a small activating RNA (saRNA) that upregulates C/EBPα, a master regulator of myeloid cell differentiation with anticancer properties. Furthermore, this saRNA restores CEBPA gene transcription, and increases both CEBPA mRNA levels and protein expression at tumor cell level activating the expression of suppressor genes that are downregulated in certain types of cancer. MTL-CEBPA has been evaluated in a phase I trial including BC patients (111). The NCT04105335 trial is now recruiting patients with solid tumors and evaluating MTL-CEBPA in combination with pembrolizumab.

MDSCs are also depleted by other new drugs like ORIN1001 that targets and binds to the RNase domain of the Inositol-requiring enzyme 1 (IRE1) involved in stress adaptation mechanisms in tumoral cells and TME (112).

### Targeting circulating macrophages and TAMs

**Figure 2:**
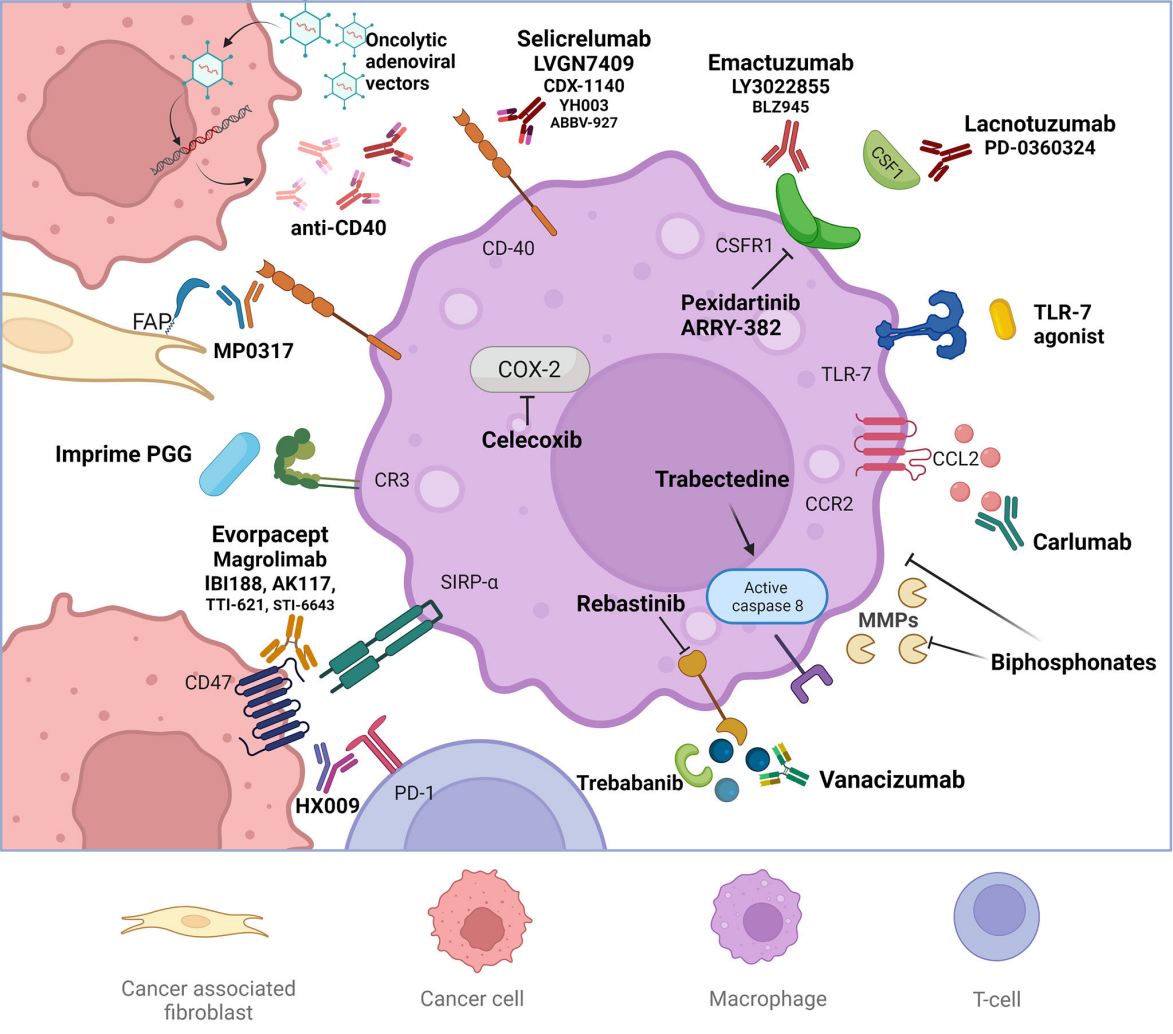
Macrophage-targeted treatment strategies on study. Macrophage-targeted treatment strategies include: inhibition of macrophage and macrophage precursors recruitment targeting the CSF1-CSFR and CCL2-CCR2 pathways, depletion of tumor associated macrophages (TAMs) (like biphosphonates), repolarization of TAMs to an antitumor phenotype, inhibition of tumorigenic factors and mechanisms promoted by TAM and enhancement of macrophage-mediated tumor cell killing or phagocytosis. The repolarization of TAMs is mediated by stimulating the costimulatory receptor CD40, Toll-like receptor 7 (TLR7) or administrating anti-CD47 drugs. Anti CR3 factors enhance the innate activity of macrophages, favoring the antitumoral phenotypes. Ang2 and the respective receptor TIE2 constitute another druggable pathway favoring antitumor responses and inhibiting the functions of TAMs.

Macrophage-targeted treatment strategies instead are showing more promising results and are currently being evaluated in many clinical trials. These strategies include: inhibition of macrophage and macrophage precursors recruitment, depletion of TAMs, repolarization of TAMs to an antitumor phenotype, inhibition of tumorigenic factors and mechanisms promoted by TAM and enhancement of macrophage-mediated tumor cell killing or phagocytosis (**Figure 2**).

The disruption of macrophage recruitment is currently being exploited targeting the CSF1-CSFR and CCL2-CCR2 pathways with specific antibodies (**Table 4**). The CCL2 blockade showed to sequester monocytes in the bone marrow, instead the inhibition of CSF1 signaling can reduce monocyte development (64, 113). Trials with novel CSF1 inhibitors showed contrasting results. The NCT01596751 trial evaluated the tolerability of PLX3397 (pexidartinib), an anti CSF1, associated with eribulin on 67 aBC patients (phase I part) and then the effect on PFS in a TNBC cohort (phase II part), but results are still awaited. Pexidartinib showed tumor response associated with paclitaxel in the BC patient group of the NCT01525602 trial (114). Conversely, lacnotuzumab, another anti CSF1, when combined to carboplatin-gemcitabine did not show a greater antitumor activity with a worse tolerability profile (115). Emactuzumab, in phase I trials including advanced BC patients, showed a specific reduction of immunosuppressive TAMs, but did not result in clinically relevant antitumor activity (116, 117). In contrast, in the NCT02265536 trial a meaningful stable disease >9 months in two patients with aBC was obtained with LY3022855 (118). However, the interruption of these treatments seems to induce a rebound effect, with abnormal elevated circulating monocytes or accelerated metastases (119).

**Table 4:** Clinical trials enrolling breast cancer patients involving new treatments targeting macrophages.

Target	Drug	Concomitant drugs	Clinical trial	Histology	Phase	Status
CSF1-CSF1R	Pexidartinib	Eribulin	NCT01596751	aBC	I/II	Completed (waiting statistical analysis)
	Pexidartinib		NCT01042379	eBC	II	Recruiting: arm closed for pexidartinib
	Emactuzumab	Atezolizumab	NCT02323191	aTNBC	I	Completed (waiting for results)
	ARRY-382		NCT01316822	aST	I	Completed (no result posted)
	ARRY-382	Pembrolizumab	NCT02880371	aST	I/II	Completed (no result posted)
	Lacnotuzumab	Spartalizumab	NCT02807844	aTNBC	I	Completed (waiting for Statistical analysis)
	PD 0360324	Avelumab	NCT02554812	aTNBC	Ib/II	Active, not recruiting
	BLZ945	Spartalizumab	NCT02829723	aTNBC	I/II	Active, not recruiting
TLR7	SHR2150	Anti-PD1, anti- CD47, chemotherapy	NCT04588324	aST	I/II	recruiting
CD47-SIRPα	Evorpacept	Pembrolizumab, trastuzumab	NCT03013218	aST	I	Active, not recruiting
	HX009		NCT04886271	aST	II	recruiting
	IBI188		NCT03717103	aST	I	Active, not recruiting
	IBI188		NCT03763149	aST	I	Completed (waiting for results)
	AK117		NCT04728334	aST	I	recruiting
	AK117		NCT04349969	aST	I	Active, not recruiting
	TTI-621	Nivolumab	NCT02663518	aST	I	recruiting
	STI-6643		NCT04900519	aST	I	recruiting
	IMC-002		NCT04306224	aST	I	recruiting
	Magrolimab	Nab-Paclitaxel, Paclitaxel	NCT04958785	aTNBC	II	recruiting
CD40	NG-350A	Checkpoint inhibitors	NCT03852511	aST	I	recruiting
	LVGN7409		NCT05152212	aST	I	recruiting
	LVGN7409	LVGN3616, LVGN3616 and LVGN6051	NCT04635995	aST	I	recruiting
	CDX-1140	CDX-301, Pembrolizumab, Chemotherapy	NCT03329950	aST	I	recruiting
	CDX-1140	Pegylated liposomal doxorubicin, CDX-301	NCT05029999	aTNBC	I	recruiting
	MP0317		NCT05098405	aST	I	recruiting
	YH003		NCT05017623	aST	I	recruiting
	YH003	YH001, Pembrolizumab	NCT05176509	aST	I	Not yet recruiting
	ABBV-927	ABBV-368, ABBV-181, carboplatin, nab-paclitaxel	NCT03893955	aTNBC	I	recruiting
	Selicrelumab	Vanucizumab, Bevacizumab	NCT02665416	aST	I	Completed (waiting for results)
	Selicrelumab	Atezolizumab, bevacizumab	NCT03424005	aTNBC	I/II	recruiting
CR3	Imprime PGG	Pembrolizumab	NCT05159778	aBC	II	recruiting
Ang2-TIE2	Trebananib	Pembrolizumab	NCT03239145	aST	I	Active, not recruiting
	Trebananib	Paclitaxel and Trastuzumab, Capecitabine and Lapatinib	NCT00807859	HER2+aBC	I	Completed (waiting for results)
	Rebastinib	Carboplatin	NCT03717415	aST	I/II	Active, not recruiting
	Rebastinib	Paclitaxel	NCT03601897	aST	I/II	Active, not recruiting
	Rebastinib	Paclitaxel, eribulin mesylate	NCT02824575	aBC	I	recruiting
COX-2	Celecoxib		NCT01881048	BC	I	Active, not recruiting
	Celecoxib	Vinorelbine	NCT00075673	BC	I	Completed (waiting for results)
MDRA	Trabectedine	Olaparib	NCT03127215	HRDt	II	recruiting

MDRA, membrane death receptors activation; LVGN3616, Anti-PD-1 Antibody; LVGN3616 and LVGN6051, CD137 Agonist Antibody; YH001, anti-CTLA-4 IgG1; ABBV-368, OX40 agonist; ABBV-181, anti PD-1, CDX-301, anti FLT3; HRDt, homologous recombination repair deficient tumors; HER2+aBC, HER2 positive advanced Breast Cancer; aTNBC, advanced Triple Negative Breast Cancer; aST, advanced Solid Tumors; COX-2, Cyclooxygenase-2.

Other treatment options that can deplete TAMs are potentially constituted by antibodies targeting antigens expressed by TAMs such as the scavenger receptor A, CD52 and folate receptor β (120, 121). However, these targets have not been studied in breast cancer models.

Bisphosphonates are also under evaluation for their capacity to induce apoptosis in monocytic cells (122). They significantly reduce complications of breast cancer bone metastasis by inhibiting resident macrophages or osteoclasts, and recent clinical trials indicate additional anti-metastatic effects outside the bone microenvironment (123). *In vitro*, bisphosphonates cause increased macrophage death whereas *in vivo* inhibit the production of pro-angiogenic factors, such as MMP-9, other evidence suggests a shifting in TAMs to a pro-tumoricidal phenotype (122).

TAMs reprogramming to a M1 phenotype can be achieved by stimulating the costimulatory receptor CD40, complement receptor 3 (CR3), administrating Toll-like receptor 7 (TLR7) and 8 agonists, inhibiting IL10 or delivering IL-12 (124). For example imiquimod, a TLR agonist, can induce the production of proinflammatory cytokines by macrophages, therefore restoring the ability to attack BC cells (125). The topical application of imiquimod showed to reduce skin metastasis in aBC patients in association to nab-paclitaxel, but responses were fleeting (126). The subcutaneous administration of a TLR agonist has been experimented in a phase II trial involving also heavily pretreated aBC patients, showing modest results and a considerable risk of cardiac toxicity (127).

Other strategies that stimulate TAMs include agonistic anti-CD40 or inhibitory anti-CD47 antibodies. The co-stimulatory receptor CD40 is expressed on macrophages and usually binds the CD154 on T cells. The agonist action of specific antibodies can reverse immune suppression and drive antitumor T cell responses (128). A first-in-human study completed in 2017 showed that the injection of a CD40 agonist antibody into superficial lesions was well tolerated and associated with pharmacodynamic responses (129). Selicrelumab, a fully human CD40 agonist, is being experimented in some phase I trials including BC patients, both alone and in association to other drugs as vanacizumab, a bispecific antibody directed to Angiopoietin 2 (Ang2) and VEGF-A (NCT02665416). Other studies are evaluating the effect of this agonist especially in TNBC, for example in association to a FMS-like tyrosine kinase 3 ligand (FLT3) inhibitor or classical chemo/immunotherapy treatments (**Table 4**). New biotechnologies targeting CD40 are also in study, such as oncolytic adenoviral vectors or designed ankyrin repeat proteins (DARPins), which are genetically engineered antibody mimetic proteins typically exhibiting a highly specific and high-affinity target protein binding. MP0317 is a DARPin intravenously administered drug targeting fibroblast activation protein (FAP) and CD40 that is currently being evaluated in a phase I trial including also aBC patients (NCT05098405).

Regarding CD47, blocking the interaction between it and the signal-regulatory protein alpha (SIRP-alfa), a “don’t eat me” signal can re-activate the phagocytic activity of TAMs (130). Many tumors overexpress CD47, enabling immune escape from the innate immune system such as macrophages binding SIRPα and compromising the antigen presentation and T cell infiltration (131). Targeting CD47 can also enhance the anti-tumor effect of other therapeutic strategies. The combination of anti-CD47 with trastuzumab, significantly suppressed the growth of antibody-dependent cellular cytotoxicity (ADCC)-tolerant HER2+ BC *via* Fc-dependent antibody-dependent cellular phagocytosis (ADCP) (132). The ASPEN-01 open-label, multicentre, phase I dose-escalation and dose-expansion study, evaluated the association of evorpacept (an anti-CD47) plus either intravenous pembrolizumab or trastuzumab. The safety findings support the use of evorpacept and preliminary data on the antitumor activity suggest future investigation. However, this trial included only one patient with aBC (133). Many other phase I and II trials are ongoing, evaluating the CD47 blocking effect on solid tumors also in aBC patients (**Table 4**). The tolerability of anti-CD47 has been successfully evaluated in a trial including five aBC patients (134).

The CD47-SIRPα axis may also be targeted using the SIRPα factor. TTI-621 (SIRPαFc) is a soluble recombinant fusion protein that acts by binding human CD47 evaluated in the. NCT02663518 trial in various solid tumors.

The macrophage-1 antigen, also called CR3, is a complement receptor consisting of CD11b (integrin αM) and CD18 (integrin β2). CR3 is a pattern recognition receptor, capable of recognizing and binding to many molecules found on the surfaces of foreign cells enabling phagocytosis (135). The clinical trial NCT02981303 have evaluated the capacity of a new molecule constituted by a pathogen-associated molecular pattern (PAMP) to enhance innate immune cell killing and the maturation of antigen presenting cells when combined to ICIs in TNBC, meeting both safety and efficacy requirements (136). CR3 is the principal β2 integrin known to contribute to PAMPs recognition (137).

There is also evidence that targeting the Ang2-TIE2 may inhibit the functions of TIE2-expressing macrophages, a TAM subset endowed with proangiogenic activity in mouse tumor models (138). Ang2 is a ligand of the TIE2 receptor and modulates endothelial cell biology facilitating angiogenesis. Ang2 inhibition, by monoclonal antibodies, peptibodies, or CovX-Bodies, may determine antitumor responses and also inhibit the functions of TAMs (139). In BC the expression of Ang2 is correlated to more aggressiveness. The intravasation occurs in sites where a TIE2-expressing macrophage and an endothelial cell are in direct contact. Ablation of the activity of these macrophages blocks intravasation after Ang2-TIE2 axis inhibition (140).

Trebananib, a peptibody that inhibits the binding of angiopoietin 1 and 2 to TIE2 showed potential anticancer effect in a phase Ib and phase II studies, with manageable AEs (141, 142). Vanucizumab is another novel bispecific antibody inhibiting VEGF-A and Ang2 that demonstrated safety and anti-tumor activity in a phase I study of 42 patients with advanced solid tumors (143). Other two trials have evaluated the tolerability of nesvacumab (an antiAng2 antibody) in advanced neoplasms (NCT01688960, NCT01271972) showing a preliminary antitumor activity (144). Rebastinib, instead, is a TIE2 inhibitor that blocks the assembly of macrophages and endothelial cells involved in metastasization at the peripheral site (tumor microenvironment of metastasis) (145). The NCT02824575 trial hypothesizes that rebastinib combined with antitubulin therapy could improve clinical outcomes in BC by preventing intravasation. The nonsteroidal anti-inflammatory drug celecoxib showed an interesting activity in BC increasing the presence of M1 like macrophages, but the real effect is in doubt (146, 147). In this perspective, the NCT00075673 trial has evaluated the weekly administration of oral vinorelbine in combination with celecoxib in aBC. Results are still awaited.

### Adoptive cell therapy

**Figure 3:**
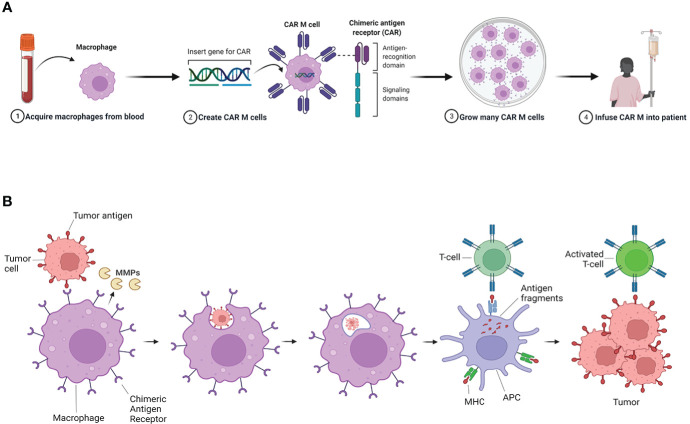
CAR-M activity in breast cancer. **(A)** Macrophages modified with Chimeric antigen receptor (CAR-M) present an improved phagocytic activity and antigen presentation capacity against tumors. CAR-M therapy is developed by the transfer of an edited specific CAR gene into macrophages withdrawn from patient peripheric blood. **(B)** These genetically modified cells are then more effective in binding to the tumor cell surface *via* specific antigen identification and active against tumor cells when reinfused into the patient. Furthermore CAR-M are able to produce metalloproteinases (MMPs) that can degrade part of the extracellular matrix (ECM) components in the tumor stroma. This activity facilitate penetration of anti-tumor immune cells into the tumor. Adapted from “Car T Cell Therapy Overview”, by BioRender.com (2022). Retrieved from https://app.biorender.com/biorender-templates.

In view of the central role of innate and adaptive immune systems in cancer development, immune cells are not only considered potential therapeutic targets, but also innovative vehicles for treatments. The genetic engineering to deliver, correct or enhance immune cells demonstrated to be successful. Chimeric antigen receptor (CAR) T-cell treatment has provided notable results in hematological tumors (148). Unfortunately, the same evidence has not been demonstrated in solid tumor, where T-cells encounter substantial difficulties in penetrating and surviving in the TME (149, 150). The extracellular matrix (ECM) is one of the major parts of TME, and it is a physical barrier to various kinds of anticancer therapies. MMPs can degrade almost all ECM components, and macrophages are an important source of MMPs (151). Amongst the cell types used in engineered cell immunotherapies, macrophages have recently emerged as prominent candidates for the treatment of solid tumors, including BC (**Figure 3**) **(** 152). In a preclinical study, macrophages engineered with specific CARs (CAR-M), activated after the detection of the HER2 antigen on tumor cell surface (153). The activation of these engineered macrophages triggered by the internal signaling of CD147 determines the production of MMPs. The infusion of CAR-147 macrophages reduces the tumor collagen deposition (153). The initial *in vitro* tests failed to show strong antitumor activity of the CAR-147, however infusion of CAR-147 cells into the aggressive HER2-4T1 bearing mouse model showed significant tumor growth inhibition (153).

One first in human phase I trial is currently active and recruiting patients with HER2+ advanced solid tumors experimenting with engineered CAR-M (NCT04660929). Another active protocol has the objective to collect tumor samples to develop patients’ derived organoids from HER2-, HER2-low and HER2+ BCs to test the antitumor activity of newly developed CAR-M (NCT05007379).

*In vitro* studies showed that CAR-M infused in tumor models increased intratumoral T-cell infiltration, NK cell infiltration, dendritic cell infiltration/activation, and TILs activation and at the same time can reduce tumor growth (152).

The combination of CAR-M treatment with other anti-tumor therapies such as CAR-T cells, ICIs and chemotherapy may synergize and provide an optimal tumor control. Nevertheless, toxicity remains an important concern and further optimization of CAR products is required (150).

Finally, preliminary outcomes about the use of mesenchimal stem cells (MSCs) in BC are interesting. Genetically modified MSCs with the insertion of tumor suppressor genes, proapoptotic genes, immune involved genes can inhibit cancer cell growth. Moreover modified MSCs delivering anticancer agents into tumor tissue have been studied in several cancer types, results in BC are awaited (154).

## Conclusions

Definitely the immune system has a very important role in cancer biology and must be taken into account when trying to understand the complexity of tumor behavior.

Increasing evidence suggests a close relationship in particular between neutrophils and macrophages with BC treatment, prognosis and outcome. Lymphocytic indexes are attractive as new potential prognostic and predictive factors for aBC treatment, mainly because they are easily detectable and applicable in daily clinical practice. Wider prospective studies are needed to unveil their real effectiveness.

The clinical efficacy of targeting immune cells (especially macrophages) in BC still needs to get official validation, but preclinical results are encouraging. Drug combination strategies seem to be the most appropriate to reduce the immunosuppressive action of immune cells in TME (155–157). The association of new compounds to classical chemotherapy, anti-HER2 agents or ICIs is currently tested in the majority of ongoing clinical trials (**Tables 3**, **4**). Combination approaches may overcome resistance mechanisms. Ongoing trials’ results are eagerly awaited to refine the optimal timing and better define treatment sequentiality to maximize therapeutic benefit.

## Author contributions

Conceptualization, CG and UD. Methodology, CG, UD and FS. Validation, UD and FS. Writing—original draft preparation, CG. Writing—review and editing, UD and FS. Supervision, UD, MP, GS, FM, CC, SB, MS, GD, SS and LC. All authors contributed to the article and approved the submitted version.

## Acknowledgments

This work was partly supported thanks to the contribution of Ricerca Corrente by the Italian Ministry of Health within the research line L2 (Innovative therapies, phase I-III clinical trials).

## Conflict of interest

This research received no external funding. MP has received advisory board fees from Novartis. UD has received advisory board or consultant fees from Merck Sharp and Dohme, Bristol Myers Squibb, Janssen, Astellas, Sanofi, Bayer, Pfizer, Ipsen, Novartis, and Pharmamar and institutional research grants from Astrazeneca, Sanofi, and Roche.

The remaining authors declare that the research was conducted in the absence of any commercial or financial relationships that could be construed as a potential conflict of interest.

## Publisher’s note

All claims expressed in this article are solely those of the authors and do not necessarily represent those of their affiliated organizations, or those of the publisher, the editors and the reviewers. Any product that may be evaluated in this article, or claim that may be made by its manufacturer, is not guaranteed or endorsed by the publisher.
